# Matrin 3 and HIV Rev Regulation of mRNA

**DOI:** 10.1186/1742-4690-8-62

**Published:** 2011-07-20

**Authors:** Andrew I Dayton

**Affiliations:** 1 FDA/CBER/OBRR/DETTD/LMV, HFM 315 1401 Rockville Pike Rockville MD 20852-1448, USA

## Abstract

The nuclear matrix protein, MATR3, is a newly-described Rev cofactor whose mechanism of action is only starting to be revealed.

## Introduction

Retroviruses by necessity have evolved mechanisms to export unspliced viral genomic and mRNA out of the nucleus and into the cytoplasm, where they can be translated or assembled into virions. The first such system discovered was the Rev/Rev Response Element (RRE) system in HIV-1. The paradigm proposed for Rev/RRE function [1], whereby interaction of Rev with the RRE (originally known as CAR, for Cis Anti-Repressor) overcomes cis repressor sequences (CRS, or INS - inhibitory sequences) that otherwise retain unspliced/partially spliced mRNA within the nucleus, has proven remarkably durable. The early debates over whether Rev acts by splicing inhibition (allowing export) or by rapid export (kinetically outracing splicing and/or degradation), have devolved to a more complex picture, in which Rev interacts with the RRE and with CRM-1/exportin 1, which in turn interacts with the phenylalanine-glycine (FG) repeats of nucleoporins, mediating export. Once in the cytoplasm, Rev-dependent mRNAs, in some systems, may also require Rev for efficient translation (reviewed in [2]). Multiple factors have been reported to interact directly or indirectly with Rev-mediated mRNA transport/expression (reviewed in [3]). Furthermore, proposals for how CRS sequences block incompletely spliced viral RNA from productive export have invoked message instability, or message retention - the latter from suboptimal splicing or binding to nuclear factors (reviewed in [4]). Whatever the mechanisms involved, the effects of the Rev/RRE interaction are only apparent when the mRNA contains CRS/INS sequences.

## Discussion

Two papers recently co-published in this journal [5,6] implicate the nuclear matrix protein, MATR3, as a co-factor used by the HIV-1 Rev protein to facilitate the nuclear export and translation of unspliced and partially spliced viral mRNA. The Marcello group came to this discovery using MS2 coat protein recognition sequence tags and MS2 pull down to survey host proteins specifically bound to HIV mRNA. The Jeang lab came to it through a yeast 2 hybrid survey of proteins that bound to PTB-1 ("polypyrimidine tract binding protein-1"), reasoning that, since PSF (a "PTB-1 associated splicing factor") inhibits the expression of CRS/INS-containing HIV-1 transcripts [7], other PTB-1 binding proteins, such as MATR3, might do so as well. Remarkably, these two divergent histories led to agreement on the facts at hand:

• MATR3 binds intracellularly to HIV mRNA.

• MATR3 has no direct effect on transcription from the HIV LTR despite its previous (though admittedly weak) implication in a role in transcription [8-11].

• For maximal function, Rev needs MATR3 to promote the cytoplasmic accumulation and (presumably consequent) translation of unspliced (or partially spliced) RRE-containing mRNA.

• Rev, MATR3 and RRE-containing mRNA form a detectable intracellular complex.

The addition of MATR3 to the pantheon of Rev cofactors is tantalizing: MATR3 exists in cells, complexed with PSF and nrbp54 [12-15]; PSF binds to CRS/INS sequences in HIV mRNA and suppresses the expression of these RNAs ([7]); MATR3 has been identified as a constituent of the nuclear pore proteome [16]. Consequently, it is tempting to view Rev as working with MATR3 to free mRNA from CRS retention (by, for example, PSF), allowing export; or as working with MATR3 to ferry mRNA to/through the nucleopore. The two mechanisms need not be mutually exclusive.

**Figure 1:**
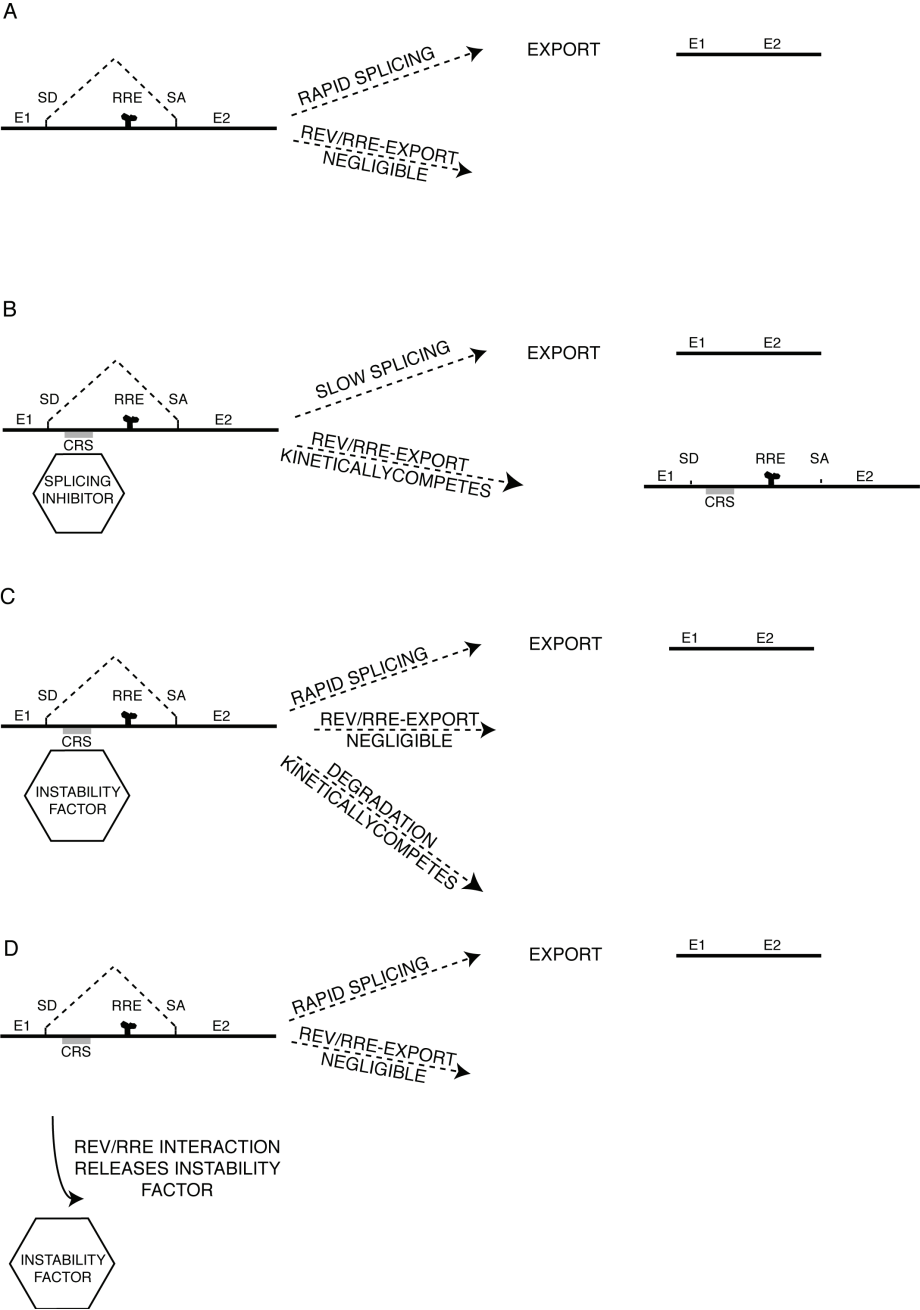
**Parsimonious kinetic models of Rev export and different mechanisms of CRS/INS function**. A) A typical cellular pre-mRNA, with typical splicing sites, even with a heterologous RRE, is not exported by Rev. B) Introduction of a "retention" or "aberrant splicing" CRS/INS into a mRNA retards splicing, allowing Rev/RRE export to effectively compete. C) Instability factors inducing degradation might be proposed to interact directly with CRS/INS, but stability alone without retardation of splicing would still not allow Rev/RRE export the opportunity to compete. D) Similarly, were Rev/RRE merely to release such an instability factor, rapid splicing would still outcompete Rev/RRE export, as in A. SD, splice donor; SA, splice acceptor; E#, exon#.

Theoretically, the pre-translational effects of Rev can be explained entirely by a simple transport mechanism based on competitive kinetics. The involvement of Rev/RRE in transport is well documented. What is important to understand is that, as mentioned above, the effects of Rev/RRE transport are only seen in the presence of CRS/INS sequences. The logical consequence of this is that any Rev mechanism must at least do more than merely abolish CRS/INS interaction with the binding partners responsible for repression. Otherwise, Rev would merely render a target mRNA equivalent to a typical mRNA which would be rapidly spliced and then exported. As depicted in Figure 1, splicing of a typical mRNA, even with an RRE, is too rapid for Rev to interdict (Figure 1A). Any CRS/INS mechanism which makes export of mRNA Rev-dependent must necessarily retard splicing (Figure 1B) to allow Rev-export. Though degradation may very well result from delayed splicing, any CRS mechanism based totally on mRNA instability can not work, because elimination of the instability would still allow rapid splicing to outrace Rev-export (Figure 1, C and 1D). Nevertheless, though parsimony does not require more than export to be involved in Rev overcoming CRS/INS inhibition and promoting cytoplasmic expression of unspliced mRNA, there is nothing to say that a multifunctional protein such as Rev does not also get directly involved in prying mRNA from various interacting partners which would otherwise contribute to its retention, such as components of aberrant splicing complexes. How MATR3 fits into any of these scenarios remains to be determined.

Finally, MATR3 has also been identified as interacting with the molecular chaperone cluster implicated by siRNA screening to be involved in viral assembly [17]. This could underlie observations that Rev is involved in encapsidation (see [18] and [2]).

## Conclusions

As is generally the case, the answers to all of these questions will emerge as more information is obtained concerning Rev/RRE and CRS/INS associated factors and their normal cellular roles. At the age of 24, the field of Rev remains rich and productive.

## Competing interests

The author declares that they have no competing interests.
